# Enamel changes of bleached teeth following application of an experimental combination of chitosan-bioactive glass

**DOI:** 10.1186/s12903-024-04195-9

**Published:** 2024-04-12

**Authors:** Farnoosh Fallahzadeh, Fahimeh Nouri, Ensiyeh Rashvand, Soolmaz Heidari, Farhood Najafi, Negar Soltanian

**Affiliations:** 1 Dental Caries Prevention Research Center, Qazvin University of Medical Sciences, Shahid Bahonar Boulevard, Qazvin, Iran; 2 Department of Operative Dentistry, School of Dentistry, Alborz University of Medical Sciences, Golshahr, Karaj, Iran; 3 Department of Resin and Additives, Institute for Color Science and Technology, Tehran, Iran; 4 Department of Restorative Dentistry, School of Dentistry, Urmia University of Medical Sciences, Urmia, Iran

**Keywords:** Chitosan, Bioactive glass, Dental Bleaching, Remineralization

## Abstract

**Background:**

Considering the extensive use of bleaching agents and the occurrence of side effects such as enamel demineralization, this study aimed to assess the enamel changes of bleached teeth following the experimental application of chitosan-bioactive glass (CH-BG).

**Methods:**

In this in vitro study, CH-BG (containing 66% BG) was synthesized and characterized by Fourier-transform infrared spectroscopy (FTIR) and X-ray diffraction (XRD). Thirty sound human premolars were bleached with 40% hydrogen peroxide, and the weight% of calcium and phosphorus elements of the buccal enamel surface was quantified before and after bleaching by scanning electron microscopy/ energy-dispersive X-ray spectroscopy (SEM, EDX). Depending on the surface treatment of the enamel surface, the specimens were divided into three groups (*n* = 10): control (no treatment), MI Paste (MI), and CH-BG. Then the specimens were stored in artificial saliva for 14 days. The SEM/EDX analyses were performed again on the enamel surface. Data were analyzed by one-way ANOVA and Tukey’s test and a p-value of < 0.05 was considered statistically significant.

**Results:**

In all groups, the weight% of calcium and phosphorus elements of enamel decreased after bleaching; this reduction was significant for phosphorus (*p* < 0.05) and insignificant for calcium (*p* > 0.05). After 14 days of remineralization, the weight% of both calcium and phosphorus elements was significantly higher compared to their bleached counterparts in both MI and CH-BG groups (*p* < 0.05). Following the remineralization process, the difference between MI and CH-BG groups was not significant (*p* > 0.05) but both had a significant difference with the control group in this regard (*p* < 0.05).

**Conclusions:**

The synthesized CH-BG compound showed an efficacy comparable to that of MI Paste for enamel remineralization of bleached teeth.

## Background

Bleaching is a minimally invasive procedure to improve tooth color. Controversy exists regarding the side effects of bleaching treatment on tooth structure. Although some studies did not find any significant effect of bleaching on tooth structure [1–4], many others reported changes in morphology, chemical composition, surface roughness, and microhardness of enamel and dentin following bleaching [5–7].

Many different remineralizing agents have been used to eliminate the adverse demineralizing effects of bleaching agents [8–13]. Bioactive glass (BG) is a biocompatible remineralizing agent that can induce cell signaling for tissue regeneration. The 45S5 BG with a composition of 46.1 mol% SiO_2_, 24.4 mol% Na_2_O, 26.9 mol% CaO and 2.6 mol% P_2_O_5_ is among the most important types of BG. Over 40 years of investigations on BG by different research teams yielded no other BG formulation with superior biological properties to 45S5 BG [14]. Upon placement in an aqueous medium, BG immediately starts its surface reactions in three phases: release and exchange of cations, dissolution of silica network, and deposition of calcium and phosphate for the formation of an apatite layer. BG has shown promising results in tooth remineralization [15, 16], and its remineralizing efficacy after bleaching treatment has been the topic of some investigations [11, 12].

Several materials such as saline, phosphoric acid, and natural and synthetic polymers have been proposed for addition to BG powder to create a paste- or gel-like consistency for easier application [17–19]. Such additives do not chemically react with BG, and their hardening only occurs through dehydration, physical reaction, or change in a component such as cross-linking of the polymer component [19]. Chitosan (CH) is a natural polymer used in combination with BG to create a paste-like or gel-like consistency [20].

CH is a polysaccharide extracted from chitin (through a deacetylation process), which is procured from natural sources such as the hard shell of crustaceans such as shrimp, crab, and lobster. Chemically, CH is a co-polymer composed of N-acetyl glucosamine and glucosamine units and has several applications due to its favorable antibacterial activity and biocompatibility [21]. The optimal efficacy of CH for remineralization of enamel defects and white spot lesions has been confirmed in several studies [22, 23]. Thus, considering the optimal efficacy of CH and BG in enamel remineralization, this study aimed to assess enamel changes of bleached teeth following the experimental application of CH-BG. The null hypotheses of this study are as follows: (1) There is no difference in the concentrations of calcium and phosphorus elements in tooth enamel before and after bleaching. (2) There is no difference between control, MI paste and CH-BG groups in terms of calcium and phosphorus elements concentrations after remineralization.

## Materials and methods

### Synthesis of CH-BG

To synthesize 45S5 BG powder by the melting method [19], the primary ingredients including SiO_2_, Na_2_CO_3_, CaCO_3_, and P_2_O_5_ (Merck KGaA Darmstadt, Germany) in certain concentrations (Table 1) were mixed with acetone in a planetary ball mill by the wet technique at 200 rpm for 2 h and were then dried at 70 °C for 2 h. The obtained powder was melted in an alumina crucible at 1400 °C for 2 h, and decarbonation was performed at 950 °C for 5 h. The melted material was quenched on the metal surface to rapidly cool down at room temperature and form amorphous glass. Finally, the obtained frit was ground in a planetary ball mill at 250 rpm to obtain BG powder.

**Table 1:** Type and amount of ingredients used for the synthesis of 45S5 BG

Type of Bioglass	SiO_2_		CaO		Na_2_O		P_2_O_5_	
	mol%	Wt.%	mol%	Wt.%	mol%	Wt.%	mol%	Wt.%
45S5	46.1	45	26.9	24.5	24.4	24.5	2.6	6

To confirm the synthesis of 45S5 amorphous glass, the obtained powder underwent X-ray diffraction (XRD; X′Pert PRO MPD, PANalytical, Netherlands) with 40 Ma, 40 kV, 2Ѳ range 10–110°, and step size: 0.02.

Next, the polymer component containing CH was synthesized. For this purpose, three solutions with certain weight and volume ratios were prepared as follows:

First solution: 2 mL of acetic acid was added to 200 mL of water to obtain an acidic solvent. Next, 2 g of CH powder was dissolved in the acidic solution at 50 °C for 2 h.

Second solution: 40 g of beta-glycerol phosphate was dissolved in 60 mL of distilled water at room temperature.

Third solution: 0.45 g of hydroxyethyl cellulose was dissolved in 40 mL of distilled water at 50 °C for 1 h. All products were purchased from Merck KGaA (Darmstadt, Germany) and Sigma Chemical Co. (St. Louis MO, USA).

Finally, the abovementioned solutions were stored at 4 °C for 12 h and were then mixed at room temperature in the following ratios to obtain a homogenous polymer liquid containing CH (final CH solution): 4 units of the first solution, 2 units of the second solution, and 1 unit of the third solution [24].

**Fig. 1:**
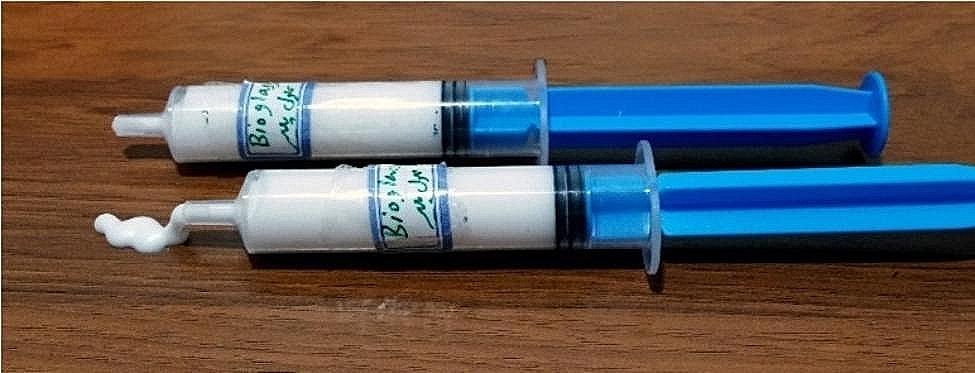
Synthesized CH-BG in a syringe

To synthesize the experimental CH-BG compound, the final CH solution was mixed with BG powder in a 33 to 66 weight ratio in a mortar and pestle at room temperature for 15 min by applying heavy manual force to obtain a homogenous flowable solution that could be easily injected by a needleless syringe and was packed in a syringe. This ratio was chosen following several trials and errors based on the best rheology in which an injectable cement is achieved. (Fig. 1)

Finally, to confirm the formulation of the synthesized product, Fourier-transform infrared spectroscopy (FTIR) was performed on three samples including BG powder, polymer solution containing CH, and experimental CH-BG compound. For this purpose, all three samples were first dried at room temperature to convert into a powder. Next, the powders were mixed with potassium bromide (KBr) in proper amounts. Subsequently, thin discs were fabricated from the materials by a pressing machine and underwent FTIR in Perkin Elmer spectroscope (Waltham, MA, USA). The transmittance spectra were reported with a wavenumber resolution of 4 cm^− 1^, in the range of 400–4000 cm^− 1^.

### Dental Specimen preparation

Based on the parameters of Coceska et al.‘s article [11], considering alpha = 0.05 & beta = 0.2, the volume of samples was calculated using Gpower software. Therefore, thirty sound human premolars extracted for orthodontic reasons were used in this study. This was approved by the Ethics Committee of Qazvin University of Medical Sciences on 29 Feb. 2020 under the code IR.QUMS.REC.1398.373. The teeth were cleaned from debris by an ultrasonic instrument, rinsed, and disinfected by immersion in 0.5% chloramine T solution for one week. They were then stored in distilled water until the experiment [25, 26].

The inclusion criteria were the absence of caries and restorations and the absence of hypoplastic defects or cracks on the buccal surface. The buccal surface of all teeth was analyzed by a DIAGNOdent pen (KaVo Dental, Germany), and teeth showing values between 0 and 12 were considered to be sound. The buccal surface of the teeth was then inspected under a stereomicroscope (MbC-2, Russia) at x10 magnification, and teeth with cracks or caries were excluded.

**Fig. 2:**
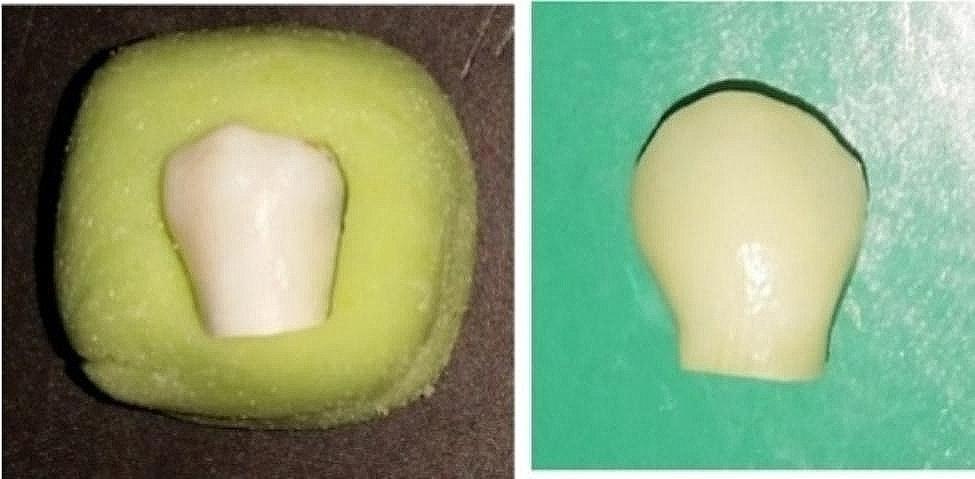
Specimen preparation

The roots were cut at 2 mm below the cementoenamel junction by a diamond disc of a cutting machine (Mecatome, Presi, France), and tooth crowns were mounted from their lingual surface in auto-polymerizing acrylic resin (Acropars200, Marlik Dental, Iran) (Fig. 2).

The specimens were randomly assigned to three groups (*n* = 10) and initially underwent energy-dispersive X-ray spectroscopy (EDX)/scanning electron microscopy (SEM, VEGA\\TESCAN-LMU, Czech Republic) to quantify the amount of calcium and phosphorus elements in the structure of enamel. The weight% of these elements was quantified at one point at the center of the buccal surface and two points 1 mm mesial and distal to the first point (a total of 3 points). The mean of the three values was calculated and reported. The parameters used for EDX included a working distance: 10 mm, accelerating voltage: 15 kV, illumination: 100 mA, and counting time: 100 s. Next, all specimens underwent bleaching within office hydrogen peroxide gel (Power Whitening YF, WHITEsmile, Germany) as instructed by the manufacturer for office bleaching. For this purpose, the bleaching agent was applied on the entire exposed facial surface with 1 mm thickness and removed with a cotton pellet after 20 min. This process was repeated three times, and a final rinse with water was then performed for 30 s. The weight% of calcium and phosphorus elements in the enamel structure was then quantified again by EDX with the same protocol as explained earlier. After bleaching, the three groups underwent a remineralization process as follows:

Control group (C): Specimens were incubated (78RH, Dorsa, Iran) at 37 °C for 14 days in artificial saliva with the composition mentioned in Table 2 with no additional intervention. The solution was refreshed daily.

MI Paste group (MI): A sufficient amount of MI Paste (GC, Tokyo, Japan) was applied on the surface of the specimens and after 5 min, it was rinsed with running water for 30 s. This process was repeated once daily for 14 days.

CH-BG group: CH-BG was applied on the surface of specimens as explained for MI Paste for 14 days.

The specimens in both M and CH-BG groups were stored in artificial saliva in between the product applications and incubated at 37 °C. The solution was refreshed daily. Finally, the weight% of calcium and phosphorus elements in the enamel structure was quantified again by EDX as explained earlier and recorded.

The normal distribution of the data was confirmed by the Shapiro–Wilk test. Data were analyzed by one-way ANOVA and Tukey’s test using SPSS version 23 at a 0.05 level of significance.

**Table 2:** Composition of materials used in this study

Material	Manufacturer	Compositions	LOT number
Power Whitening YF	WHITEsmile, Germany	hydrogen peroxide (32%), polyglycol, organic amines, silicon dioxide	19,302
MI paste	GC Corporation, Japan	pure water, CPP-ACP, D-sorbitol, propylene glycol, silicon dioxide, titanium dioxide, xylitol, phosphoric acid, flavoring, zinc oxide, sodium saccharin, ethyl p-hydroxybezoate, magnesium oxide, guar gum, propyl p- hydroxybezoate, butyl p- hydroxybezoate	181,005 C
Chitosan-Bioactive glass	experimental	Powder: bioactive glass 45S5 Liquid: Chitosan, acetic acid, beta-glycerol phosphate, hydroxyethyl cellulose	-
Artificial saliva	prepared	1.5 mmol/L Ca, 0.9 mmol/L P, 150 mmol/L KCl, and 0.05 mg F/mL in 0.1 mol/L Tris buffer, pH 7.0 [27]	-

## Results

**Fig. 3:**
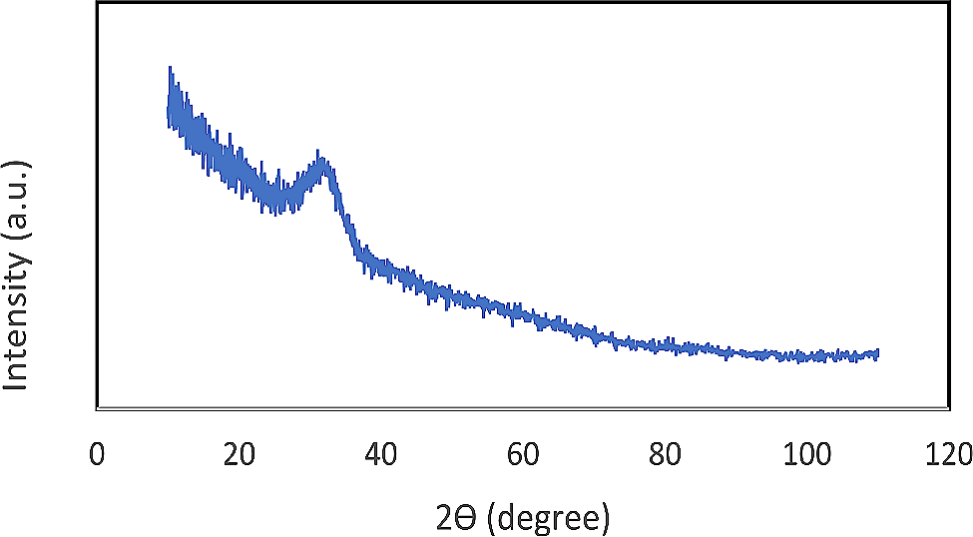
XRD spectra of 45S5 BG

According to the XRD results, the XRD spectra of 45S5 BG powder showed a broad bump at 2θ = 20°-30° indicating the amorphous structure of the synthesized material (Fig. 3).

**Fig. 4:**
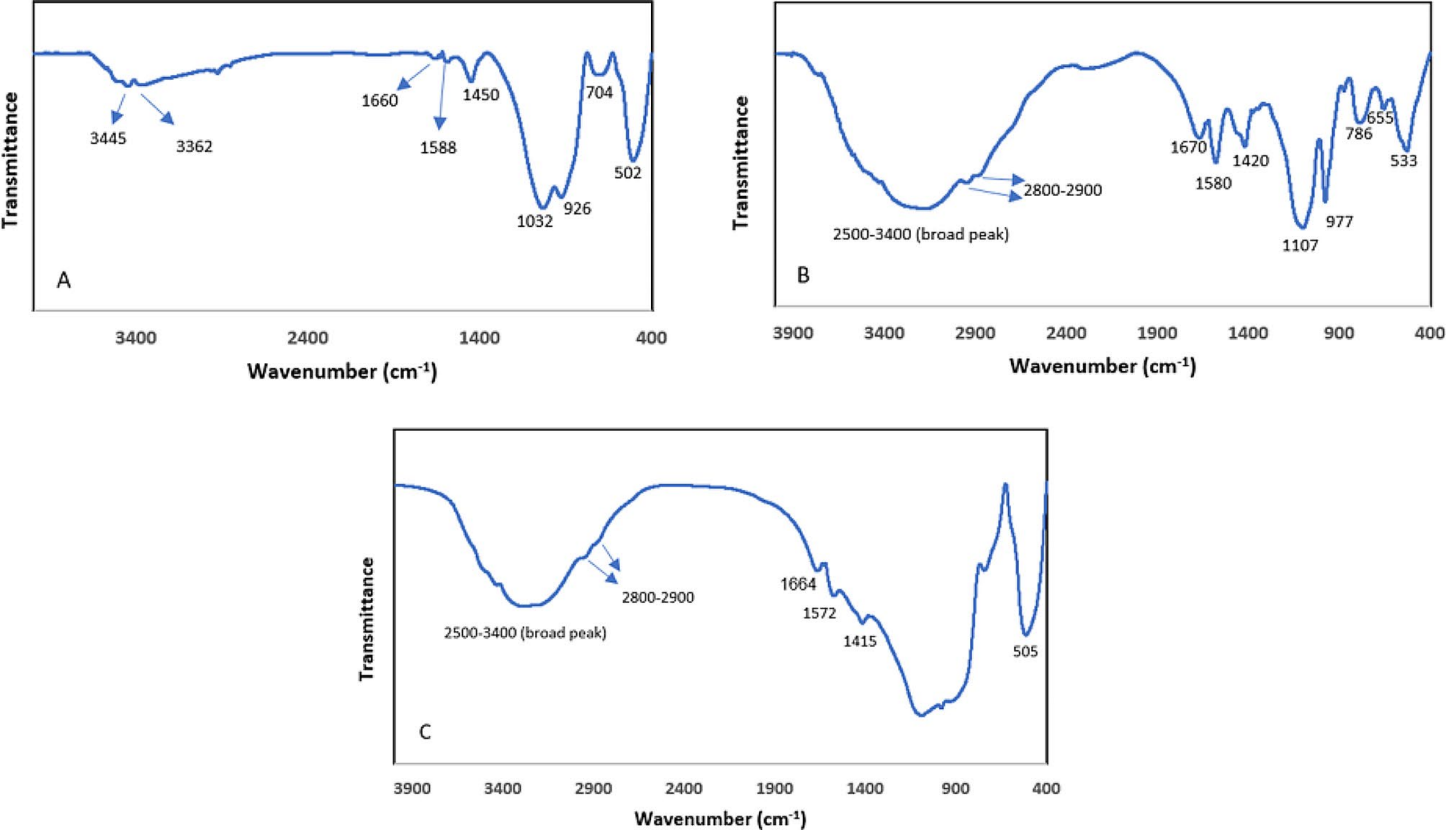
FTIR spectra of (**A**) BG, (**B**) CH solution, and (**C**) CH-BG compound

The FTIR spectra of the BG, CH solution, and final CH-BG compound are shown in Fig. 4 (A to C, respectively).

The characteristics of the FTIR spectra of BG were as follows: Si-O-Si bonds in silicate glass were noted as peaks at 1032, 926, 704, and 502 cm^− 1^. A weak peak at 1450 cm^− 1^ indicated the residual carbonate groups on the surface. The peaks at 3445, 3362, 1660, and 1588 cm^− 1^ belonged to the absorbed water molecules (O-H bonds).

The final CH solution included chitosan, beta-glycerol phosphate, and hydroxyethyl cellulose. The final functional groups in the FTIR spectra of the three compounds were as follows: stretching vibration of N-H in the amine group of chitosan at 3205 cm^− 1^, stretching vibration of N-H of the amine group of chitosan at 1580 cm^− 1^, and stretching vibration of carbonyl peak of amide in chitosan at 1670 cm^− 1^. A broad peak from 2500 to 3400 cm^− 1^ indicated the sodium salt peak related to beta-glycerol phosphate. Stretching vibration of C-O was present in the structure of chitosan, beta-glycerol phosphate, and hydroxyethyl cellulose, all appearing as a strong peak at 1107 cm^− 1^. The C-H stretching vibration of aliphatic units was noted at 2800–2900 cm^− 1^ and is related to all three compounds in the final CH solution and bending vibrations of aliphatic units appeared at 1420 cm^− 1^ is related to all three compounds in the composition of the final CH solution.

The infrared spectra of the BG-CH compound revealed a combination of peaks of both CH and BG. A peak at 505 cm^− 1^ belonged to BG and peaks at 1415, 1572, 1664, and 3252 cm^− 1^ belonged to CH bonds, probably characterizing these two components. Other peaks, due to overlap, may belong to both materials or other components. The results of EDX regarding the weight% of calcium and phosphorus in enamel structure before and after bleaching and after remineralization in the three groups are shown in Table 3.

**Table 3:** Weight% (wt%) of calcium and phosphorus in the three groups before and after bleaching and after remineralization

Element/Group	Before bleaching (baseline) Mean (wt%±SD)	After bleaching Mean (wt%±SD)	After remineralization Mean (wt%±SD)
**Ca/C**	41.69^Aa^ ± 4.39	39.70 ^Aa^ ± 4.09	39.81 ^Aa^ ± 1.37
**Ca/MI**	40.87 ^ABa^ ±2.91	39.63 ^Aa^ ± 3.97	44.42 ^Bb^ ± 4.0.6
**Ca/CH-BG**	41.44 ^ABa^ ± 3.10	39.74 ^Aa^ ± 4.11	43.62 ^Bb^ ± 3.72
**P/C**	20.07 ^Aa^ ± 0.81	16.87 ^Ba^ ± 3.15	16.91 ^Ba^ ± 1.04
**P/MI**	20.19 ^Aa^ ± 1.51	16.76 ^Ba^ ± 1.99	18.95 ^Ab^ ± 2.85
**P/CH-BG**	20.12 ^Aa^ ± 0.79	16.64 ^Ba^ ± 1.96	18.88 ^Ab^ ± 1.43

Different letters in columns and rows (for each element, separately) indicate the presence of a significant difference between them (*p* < 0.05). Different uppercase letters indicate a significant difference in rows while different lowercase letters indicate a significant difference in columns.

According to the results, the weight% of calcium and phosphorus elements in the enamel was statistically similar in all three groups at baseline (before bleaching). Also, the weight% of both calcium and phosphorus elements decreased after bleaching and this reduction was significant for phosphorus (*p* < 0.05), and insignificant for calcium (*p* > 0.05). The three groups had no significant difference from each other in this regard after bleaching (*p* > 0.05).

After 14 days of remineralization, calcium and phosphorus elements were increased in all groups compared to bleached enamel. It was significant for the CH-BG and MI groups (*p* < 0.05) while it was not significant for the control group (*p* > 0.05). The weight percentages of calcium and phosphorus elements were statistically similar in MI and CH-BG groups after remineralization and both groups had a significant difference from the control group in this regard (*p* < 0.05). The weight percentages of calcium and phosphorus in MI and CH-BG groups showed no significant difference with the corresponding values before bleaching (*p* > 0.05).

**Fig. 5:**
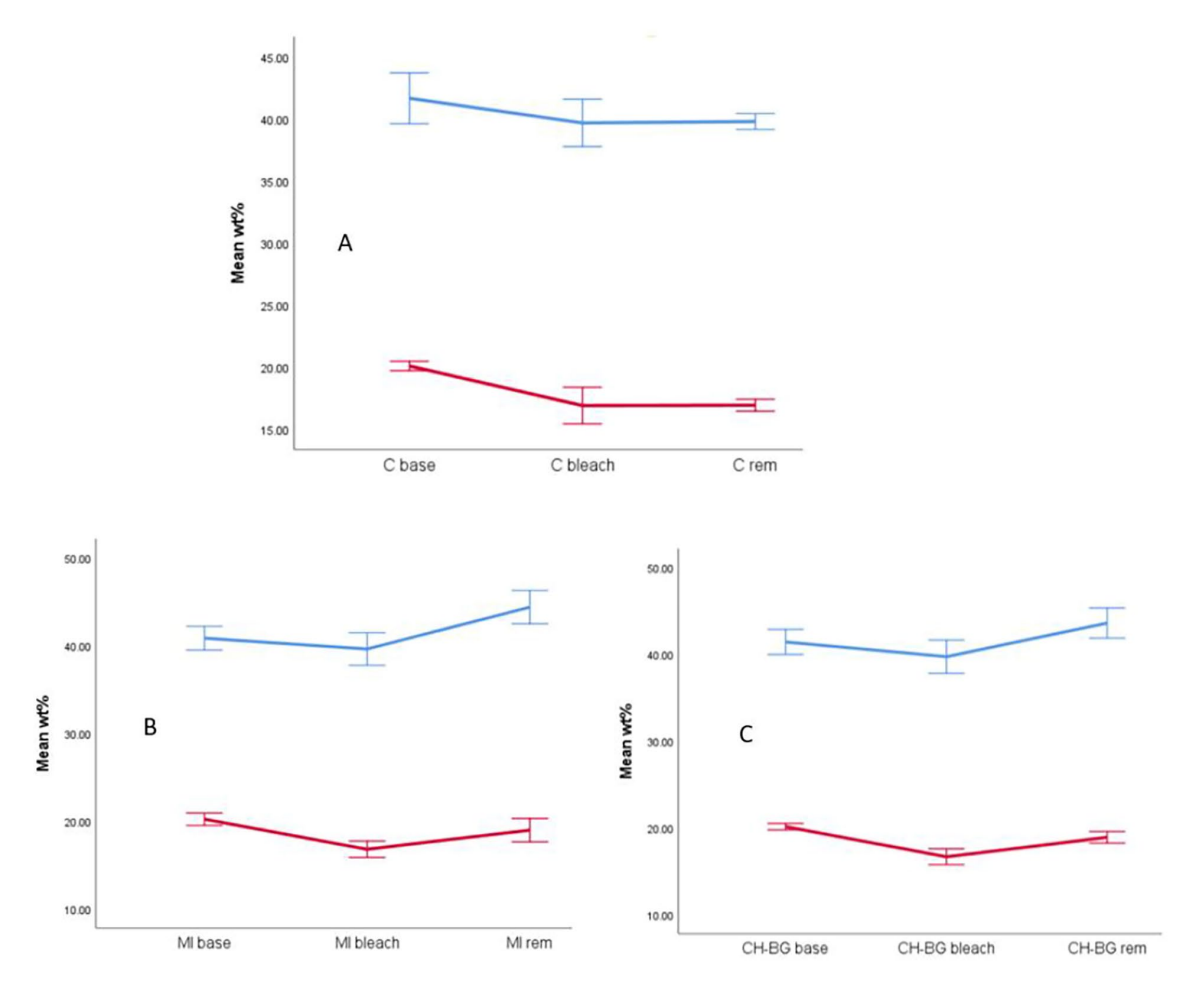
Weight percentages of calcium (blue) and phosphorus (red) elements in the three groups (**A**: Control, **B**: MI, **C**:CH-BG) at different steps (base: before bleaching, bleach, rem: after remineralization)

The weight percentages of calcium and phosphorus elements before and after bleaching and after 14 days of remineralization in the three groups are also plotted in Fig. 5 (A-C).

## Discussion

Although dental bleaching is commonly performed as a conservative procedure, controversy still exists regarding its possible adverse effects and complications, and such complications remain a topic of research [28].

Several factors have been evaluated to analyze the effect of bleaching on tooth structure; the most addressed parameters include the microhardness and mineral content of enamel. Changes in enamel microhardness, as detected by a microhardness test, can be related to the mineral content of tooth structure [5]. Nonetheless, it cannot be considered precisely equal to the change in mineral content because microhardness may be influenced by a combination of demineralization and degradation of the enamel organic matrix [6]. Since the mineral content of enamel and dentin has been suggested as a good indicator of the process of demineralization/remineralization in dental research [29], EDX was used as a complementary test in the present study to analyze the changes in the weight% of enamel calcium and phosphorus following the application of bleaching and remineralizing agents. Based on the results obtained, only the amount of phosphorus content was changed following the bleaching procedure. Therefore, the first null hypothesis was partially rejected. The EDX results revealed that despite the presence of a significant difference in the weight% of phosphorus after bleaching and after 14 days of remineralization compared with baseline in all three groups, the weight% of calcium did not experience a significant change in any group, which was in agreement with the results of Ozdemir et al. [28]. However, literature is controversial regarding the mineral content of tooth structure following bleaching. Some studies reported a significant reduction in both calcium and phosphorus contents after bleaching [9, 28] while some others reported no significant change in this regard [1, 30]. Junior et al. [31] reported a reduction in calcium and an increase in phosphorus content after bleaching [31]. Controversy in the literature in this respect can be due to the presence of a number of confounders.

Two main reasons have been proposed for complications such as reduction in microhardness and mineral content of enamel after bleaching. The first reason is that the main mechanism of bleaching treatment is the penetration of hydrogen peroxide into the tooth structure and the production of free radicals, which can oxidize the organic molecules and affect the inorganic molecules in the tooth structure [32]. Peroxide not only affects the enamel surface, but also causes moderate deproteination of inter- and intra-prismatic areas, and consequently affects minerals related to proteins, and leads to dental mineral loss [33]. The second suggested reason for the possible side effects of bleaching, which is believed to be more important than the first reason by some authors, is the pH of the bleaching agent [34–37]. Sulieman et al. [34] evaluated bleaching agents with different concentrations of hydrogen peroxide and found no significant difference in abrasion, microhardness, or surface topography of enamel and dentin among different groups. They concluded that even high concentrations of hydrogen peroxide had no adverse effect on tooth structure because the adverse effects of bleaching agents are due to their low pH and not the high concentration of peroxide. Some other studies reported lower adverse effects such as penetration of hydrogen peroxide to the pulp, dentin hypersensitivity, microscopic structural changes, or reduction in enamel microhardness due to the use of neutral, compared with acidic, bleaching gels [35–37].

Moreover, evidence shows that the potential of the bleaching procedure significantly increases at pH values > 6 and reaches its maximum level at a pH of 9. This finding is explained by the fact that an alkaline environment is critical for the optimal efficacy of bleaching agents. In alkaline environments, H_2_O_2_ more easily breaks and produces higher amounts of OH and HOO free radicals following ionic reactions. Such free radicals are active agents in the bleaching process and cleave large molecules of pigments into smaller molecules [37]. However, it should be noted that although the erosive effect of bleaching agents is minimized in neutral and high pH values, their oxidative effect remains high. Thus, since the reaction of free radicals is non-specific, organic matrix degradation is almost inevitable [6]. Therefore, Power Whitening YF (WHITEsmile) bleaching agent with a pH of 8-9.7 was selected for the present study as the most easily accessible material in terms of pH. According to the abovementioned studies, this bleaching agent can yield the best bleaching efficacy with no etching effect or erosion. Therefore, in case of occurrence of side effects and complications, they are probably due to the function of hydrogen peroxide and its effect on components of the enamel structure and not due to erosion. Resultantly, no significant reduction in calcium content after bleaching in the present study may be attributed to the high pH of the bleaching agent and no erosive effect.

Aside from pH, additives added to the bleaching agents can serve as another factor affecting the mineral content of bleached enamel. Some studies reported the efficacy of the addition of calcium and fluoride to hydrogen peroxide in the prevention of morphological and hardness changes in the enamel and enhancing the resistance of bleached enamel to erosion [35, 38]. Cavalli et al. [39] reported that bleaching agents containing calcium or fluoride, activate a remineralization mechanism simultaneous with the demineralization mechanism of the bleaching agent. This parameter explains a smaller reduction in mineral content following the use of bleaching agents containing calcium and fluoride. However, such materials cannot totally prevent this reduction and slight demineralization still occurs [39]. Yang et al. [40] added BG to hydrogen peroxide and noticed that it had no adverse effect on color change and decreased the side effects of bleaching by increasing the pH from 3.5 to 5.5 in less than 1 min. They also found that increasing the amount of BG increased the pH [40]. Information regarding the composition of the Power Whitening YF (WHITEsmile) bleaching agent is limited. Its high pH compared with other products (For Power Whitening YF the pH is 8-9.7, while it’s 6.6-7 for Opalescence Boost PF, Ultradent) [30, 36] may be due to the presence of another ingredient not disclosed by the manufacturer and its effect on the trend of change in weight% of calcium and phosphorus elements.

Another reason for variable results about the trend of change in minerals especially according to EDX can be the nature of these analyses. EDX analysis shows the weight% of elements. Thus, a reduction in one element may manifest as an increase in another element [31]. Therefore, structural analysis of the bleached enamel or the bleaching agent is required to obtain more precise results. Since dental applications of CH-BG (particularly after bleaching) have not been well investigated, this study aimed to synthesize and characterize a new CH-BG compound containing 66% bioactive glass.

Unlike the control group, the application of MI paste and CH-BG on enamel could increase Ca and P content, significantly. As a result, the second null hypothesis was also partially rejected.

Aside from dental applications of CH, there has been a growing interest in using CH-BG for tooth remineralization [21, 41]. Zhang et al. [23] evaluated the effect of pretreatment with CH on the remineralization of incipient enamel lesions and found that CH, even in the presence of pellicle, can enhance the remineralizing efficacy of BG solutions on the surface and subsurface areas, and create denser subsurface areas. Moreover, they added that subsurface remineralization caused by a combination of CH and BG is due to the ability of CH to stabilize and deliver amorphous calcium phosphate (ACP) to deeper areas of the lesion to cause subsurface remineralization by the formation of a hydroxyapatite phase in deeper areas. It should be noted that they used CH and BG slurries separately, consecutively, or mixed (and not in one formulation as in the present study) [23].

Remineralization of carious lesions occurs by the delivery of calcium and phosphate ions from the environment into the enamel lesion, resulting in the deposition of minerals in demineralized enamel. Delivery of remineralizing ions deep into the lesions plays a critical role in optimal remineralization. The positive charge of CH allows its adherence to the negatively charged demineralized enamel surface and its penetration into the enamel structure. Thus, it has the potential to deliver mineral ions into the deeper parts of the lesion. CH has been introduced as a vehicle for the delivery of ions released from BG particles [42].

Chitosan stabilizes the ACP and can induce conversion to hydroxyapatite; this property reinforces its potential to induce subsurface remineralization. CH-BG can result in the formation of CH-ACP. ACP crystals are subsequently converted to hydroxyapatite. It has been shown that some ACP crystals may be crystallized to hydroxy carbonate apatite before stabilization by CH, enhancing their washout and subsequent reduction in the uptake of minerals by the surface [23]. Since CH is charged, it can form chemical bonds between the freshly deposited minerals and demineralized enamel. It seems that the chemical reaction between hydroxyapatite and CH is the result of uniform bonds between metal ions (Ca^2+^) and amino groups of CH. In fact, CH plays an important role similar to that of Type I collagen in the prevention of crystal growth in the formation of mineral tissues [23]. The results of other studies on the combination of CH with other materials such as hydroxyapatite and amelogenin have also been highly promising [43, 44]. Pini et al. [45] reported promising results of the direct addition of CH to bleaching agents for the reduction of complications with no adverse effect on tooth color change. Couto et al. [46] evaluated the application of CH-BG compounds containing 0% (control), 10%, 20%, 30%, 40%, and 50% wt% BG in bone and observed that bone-like apatite was formed following the use of all compounds containing BG. Also, the density of formed apatite increased by an increase in BG content. Thus, in the present study, the highest amount of BG possible for use with no loss in manipulative characteristics and the form of an injectable paste was used, which was 66%.

In the present study, to assess the effect of CH-BG on the remineralization of bleached teeth, the specimens in the CH-BG group (similar to those in the MI group) were allowed 14 days for remineralization. The results showed a significant increase in the weight% of calcium and phosphorus elements after the application of CH-BG, similar to the use of MI Paste. This increase was significant in both MI and CH-BG groups compared with the control group (which was only stored in artificial saliva). Many studies, similar to the present investigation, supported the efficacy of casein phosphopeptide amorphous calcium phosphate such as MI Paste in enamel remineralization after bleaching. It has been stated that the remineralizing effect of this material is mainly based on the role of CPP in the delivery of ACP and enhancing its attachment to tooth structure [47, 48]. However, some studies such as those by Kutuk et al. and Coceska et al. [8, 11] reported different results probably due to different methodologies. They applied the remineralizing agents for a shorter period of time, used a different type of bleaching agent, and adopted a different method of bleaching compared with the present study.

In the present study, the remineralizing agents were applied for 5 min daily according to previous studies. Studies on the efficacy of CPP-ACP and BG for remineralization of bleached teeth applied the remineralizing agents for 2–5 min [10, 11]. Moreover, Arnaud et al. [22] immersed the teeth in demineralizing and remineralizing solutions and reported that CH should be used for at least 60 s to achieve the best results, and longer application times did not cause a significant change in the results.

Although CH is the main component of CH-BG synthesized in the present study, some other materials in lower concentrations were also added that play important roles in the properties of CH compound in terms of successful delivery of minerals required for efficient remineralization; the most important of which include beta-glycerol phosphate and hydroxyethyl cellulose. The amine groups of protonated CH chains have a positive charge keeping the chains away from each other and maintaining the CH in solution form. By addition of beta-glycerol phosphate, this positive charge is relatively neutralized allowing the chains to approximate to each other. However, beta-glycerol phosphate only reacts with some of the protonated amine groups and some others remain unreacted to preserve bio-adhesion of the material. On the other hand, the concentration of hydroxyethyl cellulose (HEC) as a cross-linker in the compound should be precisely adjusted to allow optimal cross-linking for adhesion of hydrogel while maintaining the viscosity low enough to allow extrusion of paste from the tube or syringe [17, 49].

Finally, it should be noted that the goal behind the synthesis of this compound was to introduce a new remineralizing agent to eliminate the side effects of demineralization caused by bleaching agents and its possible application in other demineralizing conditions such as dental caries. This study was the first on CH-BG, and considering its potential, further investigations, and particularly in vivo studies are warranted to confirm or refute its efficacy.

### Limitations

The following limitations were present in this project and should be considered in further studies:

1. The present research is an in-vitro study and despite the relative simulation, it cannot completely simulate the condition of the oral environment.
2. The effect of the synthesized CH-BG on enamel should be evaluated with more details by additional analyses, such as microhardness test, micro-computed tomography, etc.
3. Evaluation of the biocompatibility of the synthesized material should be done by cellular and molecular analyses, which were not performed in this study.

## Conclusions

1. Considering the present results, in-office bleaching with hydrogen peroxide with an alkaline pH can significantly decrease the phosphorus content with no significant reduction in calcium content. The CH-BG compound synthesized in the present study showed an efficacy comparable to that of MI Paste in remineralization of bleached enamel.
2. This study was one of a few on CH-BG (containing 66% bioactive glass) as a novel remineralizing agent. Since CH-BG showed a remineralizing property comparable to that of MI Paste, it may be suitable for widespread use. However, its optimal physical, mechanical, and biological characteristics and favourable efficacy should be confirmed in future studies and clinical trials.

## Data Availability

The datasets used and analyzed during the current study are available from the corresponding author upon reasonable request.
